# Secretory breast carcinoma: A report of three cases and a review of the literature

**DOI:** 10.3892/ol.2014.2213

**Published:** 2014-06-03

**Authors:** SEUNG GEUN LEE, SEUNG PIL JUNG, HYE YOON LEE, SINILL KIM, HOON YUB KIM, INSUN KIM, JEOUNG WON BAE

**Affiliations:** 1 Breast Center, MD Hospital, Seoul, Republic of Korea; 2 Division of Breast and Endocrine Surgery, Department of Surgery, Korea University Hospital, Korea University College of Medicine, Seoul, Republic of Korea; 3 Department of Pathology, Korea University Hospital, Korea University College of Medicine, Seoul, Republic of Korea

**Keywords:** breast, carcinoma, secretory, treatment, prognosis

## Abstract

Secretory breast carcinoma is a very rare and distinct subtype of breast cancer, characterized by the presence of intracellular and extracellular secretory material. Secretory breast carcinoma has a good clinical outcome and systemic involvement is rare. The majority of studies of this tumor have been case reports or separate analyses, and due to the rarity of these tumors, it has been difficult to fully elucidate their characteristics and define optimal treatment strategies. To add to the current knowledge of secretory breast carcinoma, the present study reports three cases of secretory breast carcinoma in patients of different ages, and with different hormone receptor statuses and treatment methods. The present study identified that each patient with secretory breast carcinoma may present with different symptoms and clinical characteristics. Therefore, therapeutic options should be selected based on the overall status of the patient and the characteristics of this rare disease.

## Introduction

Secretory breast carcinoma is a rare, invasive type of breast cancer which accounts for <0.1% of all cases of invasive breast cancer (1). It was initially termed juvenile breast carcinoma by McDivitt and Stewart (2) in 1966 as they reported seven cases of secretory carcinoma which occurred exclusively in young children with an average age of nine years. Subsequent studies have reported secretory carcinoma in adults, thus the disease was re-termed secretory breast carcinoma by Tavassoli and Norris (3) based on the histopathological characteristics of the tumor, with cells containing a vacuolated cytoplasm and the presence of intracellular and extracellular secretory material (4).

Typically, secretory breast carcinomas are negative for hormone receptors and do not express human epidermal growth factor receptor (HER)-2/neu. These tumors are slow-growing and generally have a good prognosis (1). Axillary lymph node metastases and distant metastases are rarely reported (5). Unfortunately, due to its rarity, few studies have investigated the clinical characteristics of secretory breast carcinoma and there is no consensus with regard to the best treatment strategy. Therefore, the present study reports three cases of secretory breast cancer which exhibit divergent clinical characteristics and treatment methods. Written informed consent was obtained form the patient or the patient’s family.

## Case report

### Case 1

**Figure 1:**
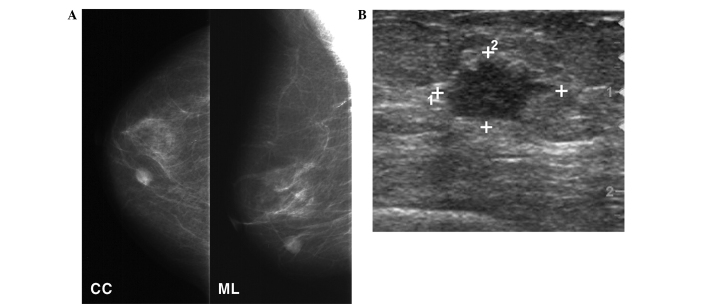
Secretory breast carcinoma in Case 1. (A) Mammography showing a round, high-density mass in the lower inner quadrant of the right breast. (B) Ultrasonography showing a 1.3-cm, speculated, irregularly shaped hypoechoic mass in the breast.

An 84-year-old female was admitted to the Department of Surgery of Korea University Hospital (Seoul, Korea) presenting with a mass in the right breast that was detected during a screening examination. The patient had no past medical history or family history of breast cancer. Mammography detected a 1.2-cm nodular density mass located in the lower inner quadrant of the right breast (Fig. 1A). Breast ultrasonography revealed a 1.3×1.0-cm, irregularly-shaped nodule in the right breast at the 5 o‘clock position, 1 cm from the nipple (Fig. 1B). During a physical examination, a 1.5-cm, well-circumscribed mass was palpated. Lumpectomy and sentinel lymph node biopsy were performed. Two sentinel lymph nodes were resected and were found to have no cancer cell involvement. Due to the patient’s age and overall health status, the patient was not treated with adjuvant chemotherapy and radiation therapy. After 61 months of follow-up, there was no evidence of breast carcinoma recurrence.

### Case 2

A 62-year-old female presented to the Department of Surgery of Korea University Hospital with bloody discharge from the right nipple for four months. The patient had a history of thyroid cancer, had undergone a total thyroidectomy approximately seven years previously and took levothyroxine sodium at a dose of 0.1 mg daily. The patient’s thyroid function test results were within the normal limits and the patient had no family history of breast cancer. Mammography revealed no suspicious lesions and the breast parenchyma was of a heterogeneous density. Breast ultrasonography revealed a 0.6-cm, round, well-circumscribed mass in the right breast at the 12 o‘clock position, 4 cm from the nipple. Lumpectomy and sentinel lymph node biopsy were performed. Three sentinel lymph nodes were found to be tumor-free. The patient received adjuvant radiation therapy with a total dose of 5500 cGy in 30 fractions and has been undergoing hormone therapy with an aromatase inhibitor. There was no evidence of disease recurrence during a 21-month follow-up period.

### Case 3

A 23-year-old female was admitted to the Department of Surgery of Korea University Hospital with a 2-cm palpable mass in the outer subareolar area of the right breast. During pre-operative breast sonography, an additional 0.8-cm irregularly shaped isoechoic mass was identified in the 4 o‘clock position in the right breast. The original 2-cm palpable mass was diagnosed as fibroadenoma, while the second 0.8-cm mass was diagnosed as secretory breast carcinoma, based on core biopsy samples. Mass excision and lumpectomy with sentinel lymph node biopsy were performed. The two harvested sentinel lymph nodes were found to be negative for cancer metastases. A total of 6,600 cGy in 33 fractions was administered to the breast. The patient also received doxorubicin- and cyclophosphamide-based chemotherapy. No relapse was observed during a 14-month follow-up period.

### Gross and histological findings

**Figure 2:**
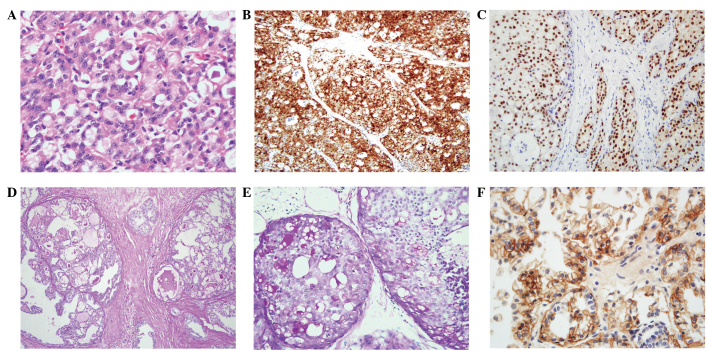
Histological findings of secretory breast carcinoma. (A) Tumor cells are large and polygonal, and possess eosinophilic cytoplasm and round nuclei. Secretory material is observed in the spaces (stain, H&E; magnification, ×400). (B) S-100 protein staining is positive in the nucleus and the cytoplasm of the tumor cells (stain, S-100 antibody; magnification, ×100). (C) Secretory materials are positive for PAS (stain, PAS; magnification, ×100). (D) PAS is positive in the secretory material in ductal carcinoma *in situ* (stain, PAS; magnification, ×200). (E) Staining for ER is positive in *in situ* and invasive ductal carcinoma (stain, ER antibody; magnification, ×200). (F) Membranous staining for EGFR (stain, EGFR antibody; magnification, ×400). H&E, hematoxylin and eosin; ER, estrogen receptor; PAS, periodic acid-Schiff; EGFR, epidermal growth factor receptor.

The tumor in Case 1 was relatively well-circumscribed. It was characterized by a multinodular, solid growth pattern with fibrous intervening stroma and was punctuated with microcystic spaces. The tumor cells were large and polygonal and exhibited abundant eosinophilic or clear cytoplasm, as well as large central nuclei with prominent nucleoli. The microcystic spaces and vacuolated cytoplasm contained densely eosinophilic secretory material, which was positive for periodic acid-Schiff (PAS), diastase-PAS (D-PAS) and Alcian blue (Table I and Fig. 2A and B). The tumor cells were found to be negative for estrogen receptor (ER), progesterone receptor (PR) or c-erbB2. Cytokeratin (CK) 5/6 and epidermal growth factor receptor (EGFR) staining were focally positive.

The tumor in Case 2 was composed of *in situ* and invasive ductal carcinoma. The ductal carcinoma *in situ* component was characterized by large, polygonal clear cells with microcystic spaces and occasional central necrosis. In the invasive areas, the tumor cell nests were within the sclerotic stroma. The spaces and cytoplasmic vacuoles contained PAS-positive and D-PAS-positive secretory material (Fig 2C and D). Staining for ER and PR was positive (Allred scores of 8 and 6, respectively; Fig. 2E) and staining for the basal markers CK5/6 and EGFR were negative. Furthermore, S-100 protein staining was observed to be negative.

The majority of the tumor in Case 3 was removed using core biopsy. The residual tumor was poorly demarcated and gray-white in color. The tumor was a mixture of macrocystic, microcystic and tubular patterns. The secretory material within the cysts, tubules and cytoplasm was positive for PAS and D-PAS. ER staining was weakly positive (Allred score, 4)and EGFR expression was observed to be positive along the cell membrane in the majority of the cells (Fig. 2F).

## Discussion

Secretory breast carcinoma is a rare and indolent type of breast tumor. Although secretory breast carcinoma was initially proposed to occur only in juvenile patients, it has been reported in patients of a wide range of ages, between 3 and 86 years old (1,6). The majority of studies have been in young females (5,7); however, Horowitz *et al* (1) reported 83 patients with a median age of 53 years obtained from the Surveillance, Epidemiology, and End Results database. The present study reported three cases of secretory breast carcinoma in patients of a wide range of ages, who presented with a variety of symptoms and clinical characteristics.

In a number of cases, secretory breast carcinoma presents as a breast mass. Bloody nipple discharge with or without a palpable mass may be the presenting form, as was observed in Case 2. Secretory breast carcinoma may resemble a benign lesion using imaging, as it may appear to be a relatively round well-circumscribed, or partially microlobulated mass with a hypoechoic or isoechoic internal echotexture (8). Reports have not shown a specific imaging pattern for secretory breast carcinoma. Thus, biopsy is essential for differential diagnosis when secretory breast carcinoma is clinically suspected.

The tumor cells in secretory breast carcinoma are polygonal with an amphophilic or clear cytoplasm. Nuclei are small with minimal atypia and low mitotic activity (4). These tumors have characteristic secretory material that may be positive for PAS, D-PAS or Alcian blue staining in both intra- and extracellular spaces. Generally, the tumors are negative for ER, PR and Her-2/neu. Certain cases express basal cell markers, including CK 5/6 and EGFR (9,10). However, there have been reports of hormone receptor-positive secretory carcinoma, consistent with the cases described in the present study (11,12).

Although rarely reported, acinic cell carcinoma, apocrine carcinoma and cystic hypersecretory carcinoma may have a cystic component or a granular cytoplasm similar to secretory breast carcinoma. With the exception of PAS and D-PAS staining, secretory breast carcinoma has similar characteristics to acinic cells, including a solid, microcystic or tubular histologic pattern, granular cytoplasm and immunoexpression of S-100 without ER expression (13). Abundant eosinophilic granular cytoplasm and gross cystic disease fluid protein 15 expression suggest that a lesion may be apocrine carcinoma. Approximately one half of apocrine carcinomas exhibit Her-2 overexpression (14), whereas the majority of secretory carcinomas are Her-2-negative (5). Unlike cystic hypersecretory carcinoma, secretory carcinoma contains only focal areas of cyst formation and produces more bubbly secretions (15). Recent studies of secretory breast carcinoma identified the balanced genetic translocation t(12;15), which generates an ETV6-NTRK3 gene fusion that differentiates secretory breast carcinoma from the ductal carcinoma, not otherwise specified (9,10). In the present study, fluorescence *in situ* hybridization (FISH) was performed in paraffin-embedded tissue sections; however, this technique failed to produce interpretable results.

Due to the limited number of reports, there is no consensus with regard to the best treatment strategy for patients with secretory breast carcinoma. These tumors are considered to be an indolent disease with an estimated disease-specific survival of >90% (1). At present, surgical excision is the primary therapy (16). Axillary metastasis is rare, particularly if the tumor is <2 cm (5). Thus, conservative treatment without lymph node examination has been frequently proposed (17). However, axillary lymph node metastasis has been reported from a 1.5-cm secretory tumor (18). Furthermore, a previous study reported that 54 out of 83 patients were diagnosed with regional lymph node metastasis (1). Involvement of more than three lymph nodes may indicate a risk for distant metastasis and a poor outcome (16). Therefore, examination of lymph node status using a sentinel lymph node biopsy or axillary lymph node dissection should be performed.

Although adjuvant chemotherapy is often administered, the use of chemotherapy has not been thoroughly investigated as secretory breast carcinoma is very slow growing and systemic metastasis is rare. Moreover, a study has reported a case that was not responsive to chemotherapy (19). In the present study, Case 3 received doxorubicin- and cyclophosphamide-based adjuvant chemotherapy, as the patient had an increased risk of recurrence due to her young age.

In general, adjuvant radiation therapy following breast conserving surgery improves locoregional control and disease-specific survival. Although there has been only one report regarding the effectiveness of radiation therapy in secretory breast cancer (1), adjuvant radiation therapy may improve long-term survival as it does for other types of invasive breast cancer. The majority of secretory tumors do not express ER, thus the effectiveness of hormone therapy has not been analyzed. The present study reported a unique case in which a tumor was strongly ER positive. The patient was treated with an aromatase inhibitor and there was no evidence of disease recurrence over a 21-month follow-up period.

In conclusion, secretory breast carcinoma is a very rare disease and there is no consensus with regard to the optimal treatment strategy. The cases described in the present study demonstrate that these tumors occur in individuals of various ages. The symptoms and clinical characteristics may also be different in each patient. Therefore, the therapeutic strategy should be selected based on the overall status of the patient and the characteristics of this rare disease.

## Figures and Tables

**Table I:** Immunohistochemical staining of secretory breast carcinoma.

Case	ER	PR	c-erbB2	CK 5/6	EGFR	S-100	c-Kit	GCDFP-15	Lysozyme	IgA
1	Negative	Negative	Score 0	Weak positive	Weak positive	Positive	Weak positive	Positive (focal/weak)	Positive	Negative
2	Positive (Allred, 8)	Positive (Allred, 6)	Score 0	Negative	Negative	Negative	Negative	Positive	Positive (focal/weak)	Negative
3	Positive (Allred, 4)	Negative	Score 0	Negative	Positive	Positive	Weak positive	Negative	Positive	Negative

ER, estrogen receptor; PR, progesterone receptor; CK, cytokeratin; EGFR, epidermal growth factor; GCDFP, gross cystic disease fluid protein; IgA, immunoglobulin A.
